# Development of a bispecific antibody that inhibits EGFR and B7H3 in NSCLC

**DOI:** 10.1186/s40364-025-00872-1

**Published:** 2025-11-25

**Authors:** Xinxin Zhi, Jiale Wang, Junhong Guo, Libo Luo, Hui Sun, Yi Li, Zhen Zhao, Chenghu Wang, Lifang Zhu, Xi Li, Feng Wang, Fei Li, Kebing Yu, Shengxiang Ren

**Affiliations:** 1 Department of Medical Oncology, Shanghai Pulmonary Hospital, School of Medicine, Tongji University, Shanghai, 200433 China; 2 Department of Pathology, Shanghai Pulmonary Hospital, School of Medicine, Tongji University, Shanghai, China; 3 Shanghai Fuhong Biopharmaceutical Co. Ltd, Shanghai, 200120 China; 4 Department of Pathology and Frontier Innovation Center, School of Basic Medical Sciences, Fudan University, Shanghai, China

**Keywords:** B7H3/CD276, EGFR, Bispecific antibody, Non-small cell lung cancer

## Abstract

**Background:**

The overexpression of epidermal growth factor receptor (EGFR) and B7 homolog 3 protein (B7H3) are known to drive the growth and proliferation of non-small cell lung cancer (NSCLC), thereby positioning them as promising therapeutic targets. This study aimed to explore the co-expression of B7H3 and EGFR in NSCLC, and thereafter develop a bispecific antibody (bsAb) targeting B7H3 and EGFR.

**Methods:**

We evaluated the co-expression of B7H3 and EGFR in 222 advanced NSCLC tissue samples, and investigated its association with the microenvironment, as well as with patient survival. Four bsAbs were designed with varying affinities to B7H3 and EGFR, and their pharmacological activities including competitive binding with EGFR, EGFR signaling blockade, and antibody-dependent cell-mediated cytotoxicity (ADCC) were evaluated. Based on the results, FH-EB02 was selected to assess the tumor growth inhibition effects with two cell line-derived xenograft (CDX) mouse models and four patient-derived xenograft (PDX) mouse models.

**Results:**

B7H3 and EGFR were co-expressed in 62.6% of advanced NSCLC and were negatively correlated with the infiltration of CD8^+^ T cells and positively associated with Foxp3^+^ Tregs. Besides, patients with positive co-expression had shorter PFS (median 7.9 vs. 14.2 months, HR = 0.50) and OS (median 17.2 vs. 37.4 months, HR = 0.47) compared to those with negative co-expression. Furthermore, by combining two anti-EGFR arms with three anti-B7H3 arms, four bsAbs were designed. The bsAbs exhibited stronger tumor cell binding capacity, more effectively EGFR signaling blockade, and more potent ADCC effects compared to cetuximab in B7H3 and EGFR double-positive cells. Among them, FH-EB02 was selected for further investigation duo to the highest binding capacity. In vivo, FH-EB02 treatment significantly inhibited tumor growth in CDX and two PDX mouse models bearing tumors coexpressing EGFR and B7H3, but showed no efficacy in PDX models expressing EGFR alone. In addition, toxicology studies in cynomolgus monkeys showed that FH-EB02 was well tolerated.

**Conclusions:**

Co-expression of EGFR and B7H3 is highly prevalent in NSCLC and correlates with poorer survival. Meanwhile, FH-EB02 is a promising B7H3/EGFR bsAb with significant anti-tumor activity and tolerable toxicities, which warrants further clinical investigation.

**Supplementary information:**

The online version contains supplementary material available at 10.1186/s40364-025-00872-1.

## Background

Lung cancer is one of the leading causes of cancer-related deaths worldwide [1, 2]. In recent years, with an in-depth understanding of the biological mechanisms of lung cancer and the development of protein engineering technologies, bispecific antibodies (bsAbs) have emerged as a novel therapeutic strategy, showing tremendous potent for prolonging the survival [3]. BsAbs have two distinct antigen-binding sites, enabling them to target two different antigens or two different epitopes of the same antigen simultaneously. This unique design allows them to play multiple roles in lung cancer treatment, including enhancing the activity of immune cells, blocking tumor cell growth signaling pathways, and reshaping the tumor microenvironment [4]. Therefore, the development of various bsAbs targeting different antigens would be crucial for further improving the therapeutic efficacy in non-small cell lung cancer (NSCLC).

The progression of solid tumors is highly dependent on the epidermal growth factor receptor (EGFR) pathway [5, 6], and cetuximab that targeting EGFR have demonstrated significant efficacy in advanced colorectal cancer and head and neck cancer [6]. As for lung cancer, in the FLEX study, patients who received chemotherapy plus cetuximab also showed significantly longer survival compared to those treated with chemotherapy-alone (11.3 months vs 10.1 months; *p* = 0.044) [7]. Furthermore, amivantamab (c-met/EGFR bsAb) has received FDA approval for three indications in EGFR-mutant NSCLC, including first-line in combination with chemotherapy for EGFR 20 insertion, first-line in combination with lazertinib, and postline in combination with chemotherapy for EGFR exon 19 deletion or L858R mutation [8–10]. Remarkably, for the patients treated with lazertinib and amivantamab, improvement in median overall survival (OS) is expected to exceed one year in the MARIPOSA study. However, EGFR is essential for the development of normal skin cells, which severe cutaneous toxicity will be induced when treated with cetuximab and amivantamab [11–14]. Therefore, an alternative strategy is needed to further enhance the efficacy of EGFR blockade and mitigate adverse effects in NSCLC.

Besides, B7 homolog 3 protein (B7H3), also known as CD276, is frequently over-expressed in various solid tumors, and low-expressed in normal tissues [15]. B7H3 played an immune-regulatory role in tumor microenvironment. B7H3 has been implicated in immune-cold tumors. Elevated B7H3 could decreased the secretion of IFN-γ, IL-2, TNF-α, and IL-13, which is important for activation and proliferation of tumor-infiltrating lymphocytes (TILs) [16, 17]. Moverover, B7H3 has been detected as an anti-inflammatory role for immune cells [18]. B7H3 promoted the differentiation of M2 macrophages through CCL2–CCR2 [19], impaired NK activation by phosphorylating and activating MYC or promoting NET formation [20]. In the complex tumor microenvironment, TILs are also subsequently reduced. Therefore, it has become a vital anti-tumor target, and its corresponding CAR-T and antibody-drug conjugates (ADC) therapies are under clinical evaluation [21, 22]. In relapsed small cell lung cancer (SCLC), B7H3-ADC achieved an objective response rate of 58.1% in ARTEMIS-001 trial and 52.4% in IDeate-Lung01 trial [23, 24]. Notably, based on ARTEMIS-001, HS-20093 has received breakthrough therapy designation from National Medical Products Administration for patients with driver gene wild-type non-squamous NSCLC, with Phase III trials are in planning. These advances demonstrate that B7H3 serves as a high-affinity target to realize specific effects on tumor cells.

It has been reported that bsAbs binding to co-expressed tumor antigens demonstrated improved tumor selectivity and reduced on-target toxicity in normal tissues [25]. Aiming to investigate the expression patterns of EGFR and B7H3, and their potential for dual blockade in NSCLC, we enrolled 222 patients from Shanghai Pulmonary Hospital, Tongji University. We further designed four bsAbs and characterized their abilities to engage with tumor cells, block EGFR signaling, and exert ADCC effects. Based on that, FH-EB02 was selected for further efficacy validation and demonstrated potent anti-tumor activity in several preclinical mouse models.

## Methods

### Enrollment of patients

Patients diagnosed with stage IIIB-IV NSCLC in Shanghai Pulmonary Hospital from January 2018 to December 2022 were continuously reviewed. The inclusion criteria were as follows: (1) ≥ 18 years of age; (2) histologically confirmed lung squamous cell carcinoma without prior systemic treatment, or EGFR/ALK/ROS1 wild-type lung adenocarcinoma without prior systemic treatment, or EGFR-mutant lung adenocarcinoma that had progressed after EGFR-TKI therapy; and (3) availability of sufficient histological specimens for immunohistochemical experiments. The study was approved by the Ethics Committee of Shanghai Pulmonary Hospital (L24-623), and informed consent forms were obtained by all enrolled patients.

### Design and production of bsAbs

BsAbs were produced in-house using the pcDNA3.1 expression vector and Expi293F cells, based on published anti-EGFR antibody sequence 2F8 of zalutumumab (WO2002100348A2), and an in-house discovered anti-B7H3 antibody. Cetuximab was produced in-house based on the published sequence (US97306598A). DE mutations (S239D, I332E) were introduced on the CH2 domain of human IgG1 Fc backbone to enhance Fc mediated antibody functions such as antibody-dependent cell-mediated cytotoxicity (ADCC). Additionally, knobs-into-holes mutations were used to facilitate correct assembly of bsAbs. BsAbs were expressed in Expi293F cells and purified using AKTA system (Cytiva, US).

### Cell lines

The NCI-H1975 cell line was acquired from the American Type Culture Collection. SK-MES-1 and A431 cell lines were acquired from the National Collection of Authenticated Cell Cultures. SK-MES-1 and NCI-H1975 cell lines with the B7H3 gene knocked out were generated in-house. NCI-H1975 was cultured in RPMI-1640 medium (Gibco, 11875093). SK-MES-1 was cultured in MEM medium (Gibco, 11095080) supplemented with 1% glutamine, 1% sodium pyruvate, and 1% non-essential amino acids. A431 was cultured in RPMI-DMEM medium (Gibco, 10566016). All the media contained 10% fetal bovine serum and were supplemented with 1% penicillin-streptomycin. All the cells were cultured in a constant temperature cell incubator at 37 °C with 5% carbon dioxide.

### Affinity measurements of bsAbs arms

Detailed binding affinity measurements of the anti-EGFR and anti-B7H3 arms of the bsAbs are provided in the online supplemental methods.

### Flow cytometry

For binding assay, NCI-H1975 cells were seeded in 96-well plate at 2 × 10^5^ cells per well and centrifugated at 600 g to remove culture media. Serially diluted bsAbs were added and incubated with cells at 4 °C for 30 minutes. After washing with stain buffer (BD bioscience, 554657, USA) to remove unbound antibodies, cells were incubated with 1:500 Alexa Fluor 488-labeled anti-human IgG secondary antibody (Jackson Immuno Research, 109–545-098, USA) at 4 °C for 30 minutes. Fluorescent signal was evaluated using flow cytometry (BD FACSLyric, USA). The median fluorescence intensity of all cells in live gate was plotted against antibody concentration in GraphPad Prism (Version 9.5.1) and EC_50_ was calculated using 4-parameter non-linear regression model. Cetuximab and IgG1 isotype antibody were used as positive control and negative control.

Fresh tissues were dissociated to generate single-cell suspension and then stained with indicated fluorochrome-conjugated antibodies in stain buffer (BD bioscience, 554657, USA). Following staining, the samples were analyzed on flow cytometer (Cytek Aurora, Shanghai, China) and analyzed using FlowJo (Version 10.9.0). The details are available in the online supplemental methods.

### Determination of EGFR and B7H3 receptor density

Receptor density on cells was reported as antibody binding capacity (ABC) and measured as described previously. Briefly, Quantum R-PE molecules of equivalent soluble fluorochrome (MESF) calibration beads and Quantum Simply Cellular anti-mouse IgG (Bangs Laboratories, Inc.) were used to determine the number of fluorophores per antibody molecule (F/P) for PE-labeled anti-EGFR antibody (Abcam, ab130738, UK) and PE-labeled anti-B7H3 antibody (BioLegend, 331605, USA). Tumor cells were individually stained with each PE-labeled antibody and analyzed in flow cytometry. The R-PE MESF beads were analyzed on the same day using identical setting to generate median fluorescence intensity vs. MESF calibration curve. MESF values for each antibody binding to the tumor cells were interpolated from the standard curve and converted to ABC based previously determined F/P ratio.

### Cell-based EGF binding blockade assay

Tumor cells were seeded in 96-well plate with serially diluted bsAbs. EGF recombinant protein (Acro Biosystems, China) pre-labeled with EZ-Link Sulfo-NHS-LC-biotin kit (Thermo Scientific, 21335, USA) was added to the cell culture at 30 ng/ml. After 30-minute incubation at 4 °C, supernatant was discarded. Cells were washed twice with PBS with 2% fetal bovine serum and incubated with Streptavidin, Alexa Fluor™ 633 conjugate (Invitrogen, USA) for 30 minutes at 4 °C. After washing and reconstitution in FACS buffer, EGF remaining on cells after antibody binding competition was detected by flow cytometry (BD FACS Lyric, USA). Cetuximab was used as positive control, while IgG isotype with or without EGF served as negative control.

### Immunohistochemistry

A total of 222 paraffin-embedded tissues were cut into 4–5 µm thick sections and placed on glass slides. The slides were then stained with primary antibodies against EGFR, B7H3, CD3, CD8, CD68, and Foxp3 at 4 °C overnight, respectively. After incubation with a secondary antibody (Servicebio, GB23301, China), the sections were observed under an optical microscope (Zeiss, Axio Scope A1, Germany). The number of positive immune cells was recorded under a 200× objective lens. The staining intensity of B7H3 and EGFR was categorized into four levels: 0, 1+, 2+, and 3+ [26]. The details are available in the online supplemental methods.

### Multiplex immunohistochemistry

Paraffin sections were dewaxed, then placed in a box filled with EDTA antigen retrieval solution and underwent antigen retrieval in a microwave oven. After autofluorescence quenching (Servicebio, G1221, China) and BSA (Servicebio, G5001, China) antigen blocking, primary antibodies, including EGFR (Servicebio, GB111504, 1:1000, China) and B7H3 (Proteintech, 66,481-1-Ig, 1:400, USA) were successively applied at 4 °C overnight. Subsequently, the Cy3 Goat anti-Rabbit IgG Antibody (Servicebio, GB21303, China) and Alexa Fluor 488 Goat anti-Mouse IgG Antibody (Servicebio, GB25301, China) were incubated in the dark at RT for 50 minutes. The nuclei were counterstained with DAPI (Servicebio, G1012, China). Finally, the slides were mounted with an anti-fluorescence quenching agent (Servicebio, G1401, China). The images were captured using an upright fluorescence microscope (NIKON, eclipse C1, Japan). The co-expression of EGFR and B7H3 was analyzed by the Image J software (version:1.54g).

### Western blot analysis

SK-MES-1 or A431 cells were cultured overnight at 2x10^5^ cells per well in 12-well plate with serum-free media. Next day, cells were incubated with 15 nM or 250 nM antibodies for 1 hour. After the antibodies incubation, 50 ng/mL EGF (Acro Biosystmes, EGF-H52H3, China) was added to stimulate cells for 15 minutes. Cells were lysed with RIPA buffer and analyzed by western blotting. Primary antibodies were used in the following condition: p-EGFR Tyr1068 (Cell Signaling Technology, 2234, USA) 1:200 dilution，EGFR (Cell Signaling Technology, 4267S, USA) 1:200 dilution. Images were acquired on Bio-Rad ChemiDoc MP (USA).

### ADCC killing assay

Cryo-preserved human PBMC from healthy donors (Oribiotech, China) were thawed and recovered after overnight incubation in RPMI 1640 complete media. 1 × 10^6^ tumor cells were labeled with 4 μL BADTA reagent from DELFIA EuTDA Cytotoxicity Detection kit (Perkin Elmer, AD0116, USA). Tumor cells and PBMC were mixed at a target to effector cell ratio of 1:25. Serially diluted antibodies were added and co-incubated for 3 hours at 37 °C. After incubation, cells were centrifugated and supernatant was transferred to a new plate to mix with Europium solution (Perkin Elmer, USA). Plates were incubated in the dark with shaking for 15 min, then time resolved fluorescence was measured on SpectraMax I3X (Molecular Devices, USA).

### Mouse experiments

BALB/c nude mice (6–8 weeks old) were purchased from Charles River (Beijing, China) and maintained under standard animal-care conditions. A total of 3.5 × 10^6^ SK-MES-1 cells or 5 × 10^6^ NCI-H1975 cells were implanted to the right hand of each mouse, respectively. After the tumors reached approximately 100 mm^3^, the mice were evenly divided into four groups (*n* = 7 per group) according to the tumor size and given different doses of FH-EB02 or PBS: 1, 5 and 20 mg/kg for NCI-H1975 models, and 1, 5 and 15 mg/kg for SK-MES-1 models. Intraperitoneal injection was performed twice a week on day 1 and day 4 for three weeks. Tumor volume and body weight were measured at three-day intervals. The formula for calculating tumor volume (V) was V = L × W^2^/2, where L denotes the length of the major axis and W denotes the length of the minor axis.

PDX studies were conducted at Shanghai Pulmonary Hospital. Following patient consent and approval from the hospital’s ethics committee, surgical tissues from 26 untreated NSCLC patients was used to establish PDX models in NOG and BALB/c nude mice aged 6–8 weeks. Tumor tissues were cut into pieces of approximately 3 mm^3^ and implanted subcutaneously on the right flank of each mouse. Once the tumors reached a volume of 1000–1500 mm^3^, the tissue was removed and implanted using the same method into a new group of mice on the right flank. After four cycles, when the tumor volume had increased to 100 mm^3^ approximately, the mice were evenly divided into control and experimental groups (*n* = 6–8 per group) based on tumor volume for drug efficacy experiments. All the animal studies were approved by the Institutional Committee for Animal Care and Use, Shanghai Pulmonary Hospital and were performed in accordance with the institutional guidelines.

### Non-human primate safety study

Non-human primate pilot toxicology study was performed at Guochen Biotechnology Co. (Suzhou, China) in accordance with standard operating procedures. Cynomolgus monkeys (*N* = 2 per dosing group, age 3 to 5 years, one female weighting 2.7–2.8 kg and one male weighting 3.0–3.2 kg) received FH-EB02 at 60 mg/kg or 100 mg/kg. FH-EB02 was administered by intravenous infusion on days 1, 8, 15 and 22, with an infusion duration of approximately 30 minutes per monkey. Clinical signs, body weight, food consumption and infusion site reaction were monitored throughout the study. Blood was collected at day 1, 3, 7, 14, 21, 29 to assess peripheral blood cell profile and blood chemistry. Serum samples were sequentially collected prior to administration of the first and last doses, and at post-administration time points of 10 minutes, 2 hours, 6 hours, 24 hours, 48 hours, 72 hours, 120 hours, and 168 hours. The concentration of FH-EB02 in serum was determined using an Enzyme-Linked Immunosorbent Assay. The accumulation index was defined as the ratio of the maximum observed plasma concentration (Cmax) on day 22 to Cmax on day 1. Drug exposure was calculated based on the area under the pharmacokinetic curve from time zero to the last time point (AUC_0-t_).

### Statistics

Statistical analyses were performed using IBM SPSS (version 26), GraphPad Prism (version 9.5.1) and R software (version 4.4.2). Comparisons between two groups of continuous variables were analyzed using Student’s t-test (two-tailed). Comparisons between two groups of categorical variables were conducted using the χ^2^ test and Fisher’s exact test. Comparisons among multiple groups of continuous variables were analyzed using One-way repeated-measures ANOVA test. PFS and OS were estimated by Kaplan-Meier method and compared the significance of difference by log-rank test. *p* < 0.05 was considered to indicate a statistically significant difference.

## Results

### Co-expression of B7H3 and EGFR in advanced NSCLC and its correlations with prognosis and tumor immune microenvironment features

**Fig. 1:**
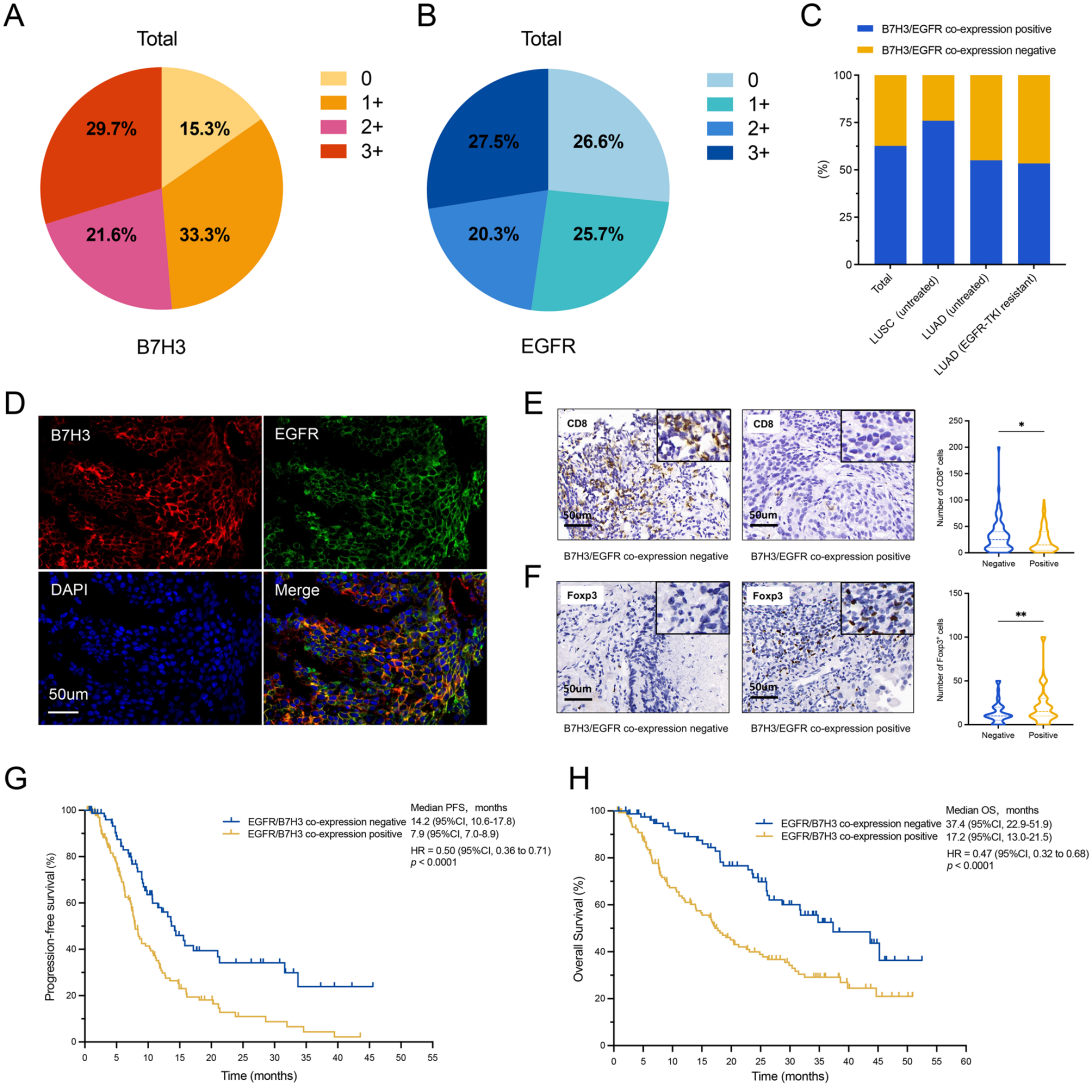
The expression of B7H3 and EGFR and their correlations with patient prognosis and immune infiltration in advanced NSCLC. **A** and **B**, distribution of expression levels of B7H3 (**A**) and EGFR (**B**) in the total population. **C**, Co-expression of B7H3 and EGFR in the indicated population, where double-positive specimens were defined as co-expression positive, and double-negative or single-positive samples were defined as co-expression negative. **D**, Representative immunofluorescence images demonstrating co-expression of B7H3 and EGFR. The histological subtype was lung squamous cell carcinoma. E and F, Representative immunohistochemical images of CD8 (**E**) and Foxp3 (**F**) staining and corresponding statistics analyses of the data. **G** and **H**, the relationships between B7H3 and EGFR co-expression and progression-free survival (**G**) and overall survival (**H**) in the total population. *p < 0.05, **p < 0.01

From January 2018 to December 2022, a total of 222 patients with stage IIIB–IV NSCLC were enrolled for analysis, classified into three subgroups: 87 untreated lung squamous cell carcinoma (LUSC) cases, 60 treatment-naïve EGFR/ALK/ROS1 wild-type lung adenocarcinoma (LUAD) cases, and 75 EGFR-mutant LUAD cases with acquired resistance to EGFR-TKI. The clinicopathological characteristics are detailed in Supplemental Table 1 and Table 1. We classified B7H3 and EGFR expression into four grades based on immunohistochemical staining intensity, with representative images provided in Supplemental Figure 1A and B. The distribution of B7H3 expression levels (0, 1+, 2+, and 3+) across the total population was 15.3%, 33.3%, 21.6%, and 29.7%, respectively (Fig. 1A; Supplemental Table 1), while EGFR expression levels were 26.6%, 25.7%, 20.3%, and 27.5% (Fig. 1B; Supplemental Table 1). The expression levels of B7H3 and EGFR in the three subgroups were given in Supplemental Figure 1C-H and Supplemental Table 1. We defined B7H3 and EGFR double-positive samples as B7H3/EGFR co-expression positive, and double-negative or single-negative samples as B7H3/EGFR coexpression negative. Positive B7H3/EGFR co-expression was observed in 62.6% (139/222) samples analyzed (Fig. 1C; Supplemental Table 1). Specifically, 75.9% untreated LUSC, 55.0% untreated EGFR/ALK/ROS1 wild-type LUAD, and 53.3% EGFR-TKI resistant LUAD showed positive co-expression (Fig. 1C; Supplemental Table 1). The multiplex immunofluorescence staining clearly demonstrated the co-expression of B7H3 and EGFR on the membrane of tumor cells (Fig. 1D).

**Table 1:** Relationship between B7H3 and EGFR co-expression and clinical features as well as the immune microenvironment in patients with advanced NSCLC

	LUSC (untreated)		*P*	LUAD (untreated)		*P*	LUAD (EGFR TKI-resistant)		*P*	Total		*P*
	Negative (*N* = 21)	Positive (*N* = 66)		Negative (*N* = 27)	Positive (*N* = 33)		Negative (*N* = 35)	Positive (*N* = 40)		Negative (*N* = 83)	Positive (*N* = 139)	
**Gender**												
Male	20 (95.2%)	65 (98.5%)	0.427	23 (85.2%)	28 (84.8%)	1.000	18 (51.4%)	22 (55.0%)	0.757	61 (73.5%)	115 (82.7%)	0.100
Female	1 (4.8%)	1 (1.5%)		4 (14.8%)	5 (15.2%)		17 (48.6%)	18 (45.0%)		22 (26.5%)	24 (17.3%)	
**Age (years)**												
Mean (SD)	66.5 (6.45)	65.5 (7.02)	0.543	63.7 (8.67)	65.2 (7.44)	0.471	63.6 (10.5)	60.0 (12.4)	0.181	64.4 (9.00)	63.8 (9.23)	0.671
**ECOG PS**												
0–1	19 (90.5%)	63 (95.5%)	0.590	25 (92.6%)	29 (87.9%)	0.681	26 (74.3%)	33 (82.5%)	0.412	70 (84.3%)	125 (89.9%)	0.218
2	2 (9.5%)	3 (4.5%)		2 (7.4%)	4 (12.1%)		9 (25.7%)	7 (17.5%)		13 (15.7%)	14 (10.1%)	
**Smoking history**												
No	5 (123.8%)	34 (51.5%)	0.710	14 (51.9%)	19 (57.6%)	0.658	29 (82.9%)	34 (85.0%)	0.801	48 (57.8%)	87 (62.6%)	0.482
Yes	16 (76.2%)	32 (48.5%)		13 (48.1%)	14 (42.4%)		6 (17.1%)	6 (15.0%)		35 (42.2%)	52 (37.4%)	
**Stage**												
III	10 (47.6%)	33 (50.0%)	0.849	8 (29.6%)	5 (15.2%)	0.176	0 (0%)	2 (5.0%)	0.496	18 (21.7%)	40 (28.8%)	0.245
IV	11 (52.4%)	33 (50.0%)		19 (70.4%)	28 (84.8%)		35 (100%)	38 (95.0%)		65 (78.3%)	99 (71.2%)	
**PD-L1**^**#**^												
< 1%	10 (47.6%)	26 (39.4%)	0.497	12 (44.4%)	15 (45.5%)	0.845	19 (54.3%)	17 (42.5%)	0.265	41 (49.4%)	58 (41.7%)	0.262
1–49%	3 (14.3%)	17 (25.8%)		5 (18.5%)	5 (15.2%)		5 (14.3%)	12 (30.0%)		13 (15.7%)	34 (24.5%)	
≥50%	5 (23.8%)	12 (18.2%)		7 (25.9%)	11 (33.3%)		9 (25.7%)	9 (22.5%)		21 (25.3%)	32 (23.0%)	
CD3*												
Mean (SD)	73.1 (33.1)	68.0 (41.3)	0.611	71.9 (49.2)	85.8 (47.8)	0.273	38.9 (31.9)	35.4 (24.2)	0.593	58.3 (41.7)	62.8 (43.1)	0.438
CD8*												
Mean (SD)	45.0 (29.8)	24.7 (22.5)	0.001	33.7 (41.4)	37.7 (26.4)	0.650	22.1 (18.2)	11.1 (11.5)	0.003	31.7 (31.4)	23.9 (23.1)	0.034
CD68*												
Mean (SD)	48.8 (27.5)	37.7 (26.4)	0.098	32.8 (31.1)	40.9 (31.9)	0.327	25.6 (23.3)	31.4 (28.2)	0.338	33.8 (28.4)	36.6 (28.3)	0.475
FOXP3*												
Mean (SD)	14.3 (11.6)	17.5 (16.6)	0.412	20.6 (16.4)	39.1 (31.2)	0.007	8.4 (7.3)	12.1 (9.9)	0.072	13.9 (13.0)	21.1 (22.1)	0.002

#Indicates that patients with missing PD-L1 immunohistochemistry data were excluded from the statistical analysis

*Indicates that the number of cells were observed under a 200× objective lens. LUSC, lung squamous cell carcinoma; LUAD, lung adenocarcinoma; SD, standard deviation

Analysis of the immune microenvironment revealed that samples with co-expression of B7H3 and EGFR had fewer CD8^+^ T cells (Fig. 1E; Table 1) and more Foxp3^+^ Treg cells (Fig. 1F; Table 1) infiltration, with no significant difference in CD3 and CD68 expression (Supplemental Figure 1I-L; Table 1). In the overall population, patients with negative B7H3/EGFR co-expression had significantly longer progression-free survival (PFS) (14.2 vs. 7.9 months, HR = 0.50 [95% CI, 0.36–0.71], *p* < 0.0001) and OS (37.4 vs. 17.2 months, HR = 0.47 [95% CI, 0.32–0.68], *p* < 0.0001) compared to those with positive (Fig. 1G and H). Detailed analyses also demonstrated similar trends in the three subgroups (Supplemental Figure 2). Overall, these findings indicated that B7H3/EGFR co-expression was highly prevalent in advanced NSCLC and correlated with poor survival and potentially impaired anti-tumor immunity, thereby underscoring the significance and promising clinical application of effective anti-EGFR/anti-B7H3 bsAbs.

### Development of anti-EGFR/anti-B7H3 bsAbs

**Fig. 2:**
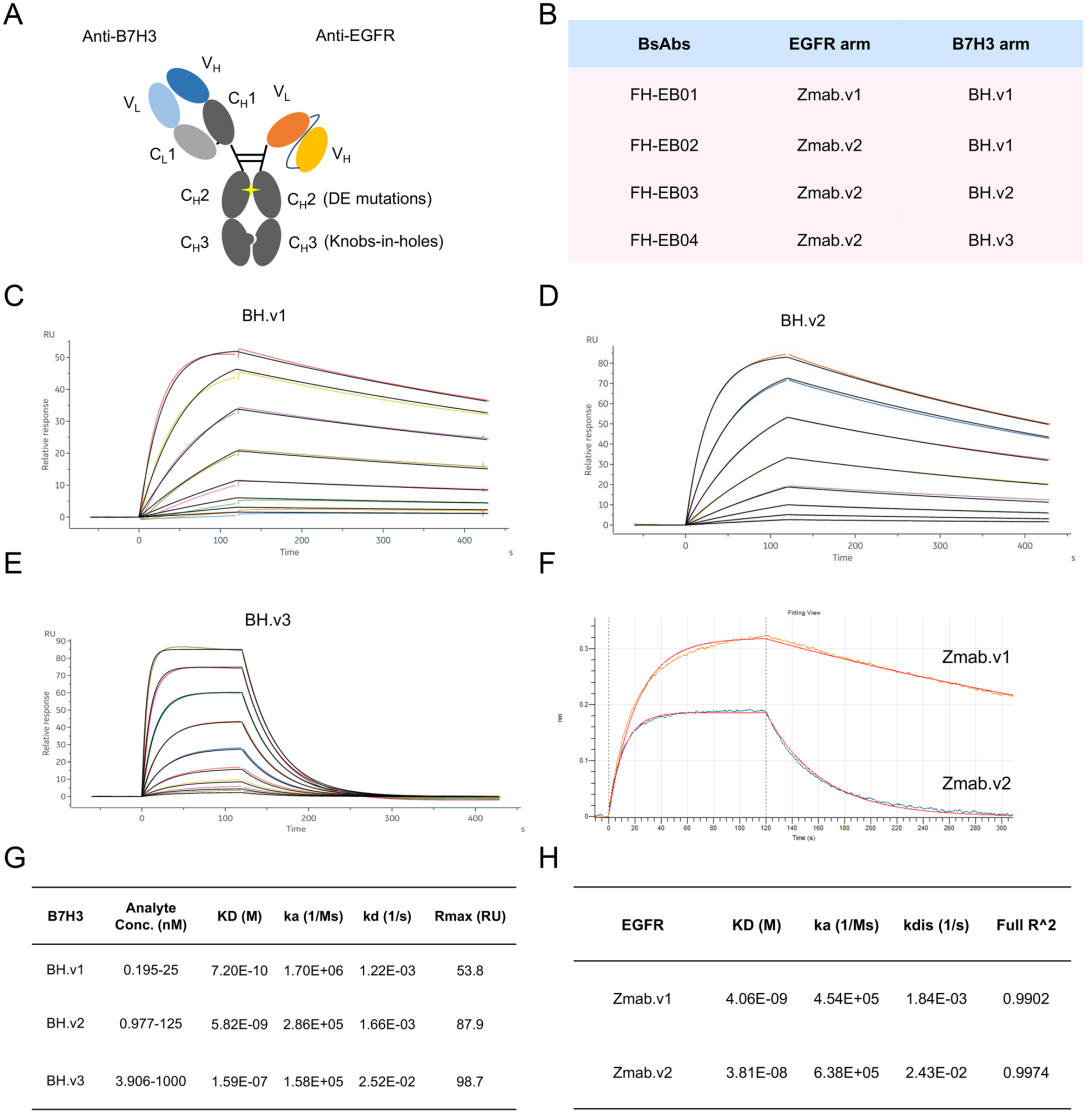
Development of B7–H3 and EGFR bispecific antibodies. **A**, schematic representation of FH-EB02. FH-EB02 is designed as a bsAb containing one anti-B7H3 arm and one anti-EGFR arm. DE mutations (S239D, I332E) were introduced into the CH2 domain, and knobs-into-holes mutations were introduced into the CH3 domain of the human IgG1 fc backbone. **B**, four bsAbs with different anti-EGFR and anti-B7H3 arms. **C-E**, affinity measurements of B7H3-binding arms, including BH.v1 (**C**), BH.v2 (**D**), and BH.v3 (**E**), using surface plasmon resonance assays. **F**, affinity measurements of EGFR-binding arms of Zmab.v1 and Zmab.v2 using biolayer interferometry (bli) assays. **F**, the results of the surface plasmon resonance experiments in (**C-E**). **H**, the results of the affinity measurement experiments in (**F**)

To selectively target EGFR and B7H3 double-positive tumors, we generated four anti-EGFR/B7H3 bsAbs by fine tuning antigen binding affinity on each arm (Fig. 2A-H). The anti-B7H3 arm was selected from anti-B7H3 Fab modules with a monovalent binding affinity of 7.20E-10 M (BH.v1), 5.82E-09 M (BH.v2) or 1.59E-07 M (BH.v3) to human B7H3 as measured by Surface plasmon resonance (Fig. 2C-E and G), whereas the anti-EGFR arm was selected from single chain fragment variable (scFv) modules derived from Zalutumumab, with a monovalent binding affinity of 4.06E-09 M (Zmab.v1) or 3.81E-08 M (Zmab.v2) to human EGFR as measured by Biolayer Interferometry (Fig. 2F and H).

### The bsAbs selectively bound to tumor cells coexpressing EGFR and B7H3 and inhibited the EGFR signaling pathway

**Fig. 3:**
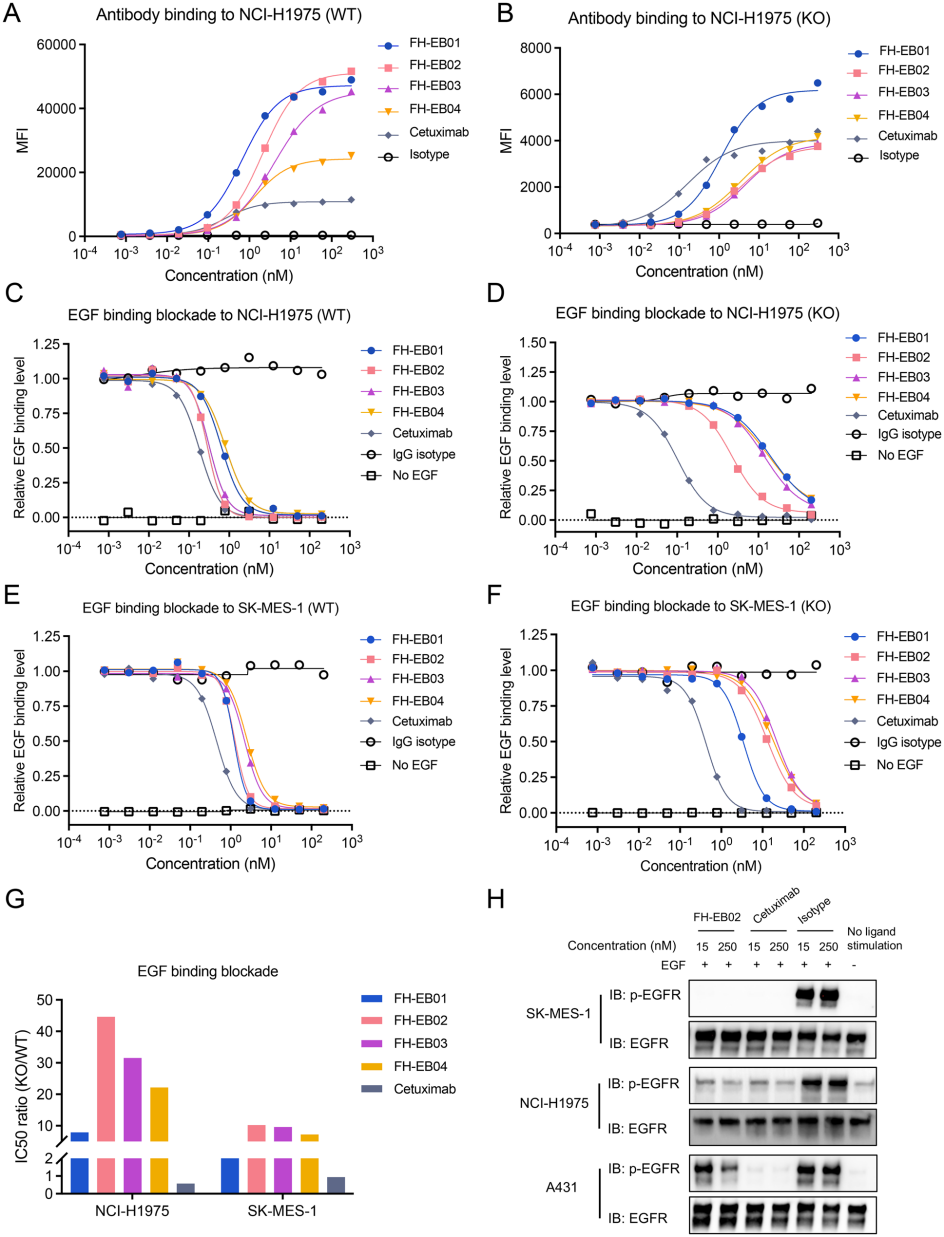
The bsAbs competitively bound with EGFR and inhibited EGFR signaling in B7H3 and EGFR coexpressing NSCLC cell lines. **A** and **B**, dose-dependent binding profiles of the four bsAbs with wild-type (**A**) or B7H3-knockout NCI-H975 cells (**B**). **C**-**F**, competition binding assays of the bsAbs and EGF ligand to wild-type (**C**) or B7H3-knockout (**D**) NCI-H1975 cells, wild-type (**E**) or B7H3-knockout (**F**) SK-MES-1 cells. **G**, the ratio of IC50 (KO) to IC50 (WT) accessed in (**C-F**). **H**, inhibition of the EGFR signaling pathway by FH-EB02 or cetuximab in SK-MES-1, NCI-H1975 and A431 cells. WT, wide type; KO, B7H3-knockout

We subsequently evaluated the binding capacity of these bsAbs in NCI-H1975 cells (EGFR-mutant LUAD cell line) that are double-positive for EGFR and B7H3 (Supplemental Figure 3A), as well as in its isogenic single-positive cells (B7H3 knockout) expressing only EGFR. The binding features of bsAbs to NCI-H1975 wide type (WT) cells varied among the four bsAbs with differentiated anti-EGFR and anti-B7H3 arms (Fig. 3A). All bsAbs achieved a higher binding capacity than the monospecific anti-EGFR antibody cetuximab (Fig. 3A). Notably, FH-EB02, which incorporated an anti-EGFR arm with a lower affinity and an anti-B7H3 arm with the highest affinity, showed greater binding capacity (Fig. 3A). To figure out whether the binding depends on B7H3 expression, we further tested the binding capacity in NCI-H1975 B7H3 knockout (KO) cells. We observed that all four bsAbs exhibited significantly reduced binding capacity compared to their binding to NCI-H1975 (WT) cells (Fig. 3B). In contrast, cetuximab showed no substantial difference (Fig. 3B). Collectively, we found that the four anti-EGFR/anti-B7H3 bsAbs had greater binding affinity to tumor cells compared with cetuximab, especially FH-EB02. Importantly, this binding greatly relied on the expression of B7H3, highlighting the potential of these bsAbs to mitigate off-target toxicities associated with cetuximab’s binding to EGFR on normal cells.

Inhibition of the EGFR signaling pathway through ligand blockade represents a critical mechanism of action for EGFR-targeted antibody therapeutics [5]. We subsequently evaluated the capacity of bsAbs to impede EGF ligand binding to EGFR expressed on tumor cells. Both NCI-H1975 (WT) and NCI-H1975 (KO) cells were pre-treated with antibodies or isotype controls before exposure to EGF. The amount of cell-bound EGF was quantified using flow cytometry. All bsAbs exhibited robust blockade of EGF binding in NCI-H1975 (WT) cells (Fig. 3C). In NCI-H1975 (KO) cells, the inhibitory effects of these bsAbs were significantly impaired (Fig. 3D). Consistent results were observed in SK-MES-1 cell line (B7H3/EGFR co-expressing LUSC cell line, Fig. 3E, Supplemental Figure 3A) and its isogenic B7H3 knockout counterpart (Fig. 3F). The IC50 ratios of NCI-H1975 (KO) to NCI-H1975 (WT) were 7.8, 44.6, 31.6, and 22.2 for FH-EB01, FH-EB02, FH-EB03, and FH-EB04, respectively (Fig. 3G). In the SK-MES-1 model, the corresponding IC50 ratios were 2.9, 10.2, 9.6, and 7.3 for FH-EB01, FH-EB02, FH-EB03, and FH-EB04, respectively (Fig. 3G). FH-EB02 showed greater effect on NCI-H975 cells compared to SK-MES-1 cells (Fig. 3G). Besides, in both instances, FH-EB02 had the highest IC50 ratio, consistent with its weaker anti-EGFR arm and stronger anti-B7H3 arm (Fig. 2G-H, and 3 G). In contrast, although cetuximab potently blocked EGF ligand binding, it lacked selectivity between WT and KO cells (Fig. 3C-G). These findings suggested that these bsAbs, especially FH-EB02, may selectively target tumor cells co-expressing both EGFR and B7H3, while sparing cells that express only EGFR.

The binding of EGF ligand to its receptor EGFR can activate the EGFR signaling cascade [5]. We assessed EGFR phosphorylation in cells stimulated with EGF in the presence of FH-EB01, FH-EB02, FH-EB03 or cetuximab on NCI-H1975, SK-MES-1 and A431 cell lines (Supplemental Figure 3B). A431 was an epidermoid carcinoma cell line representing normal skin with significant EGFR overexpression and relatively low expression of B7H3 (Supplemental Figure 3A). In both NCI-H1975 and SK-MES-1, both 15 nM and 250 nM of FH-EB01 and FH-EB02 effectively inhibited EGF-stimulated EGFR phosphorylation to a similar extent as cetuximab (Fig. 3H, Supplemental Figure 3B). FH-EB03 showed a weaker inhibition of EGF-stimulated EGFR phosphorylation (Supplemental Figure 3B). In the A431 cell line, all three bsAbs exhibited weaker inhibition of EGF-stimulated EGFR phosphorylation compared with cetuximab (Supplemental Figure 3B). Besides, the inhibitory effect of FH-EB02 on phosphorylated EGFR (p-EGFR) was considerably weaker even at 250 nM (Fig. 3H), indicating selective inhibition EGFR signaling in tumor cells co-expressing EGFR and B7H3.

### BsAbs mediate more potent ADCC in B7H3 and EGFR coexpressing tumor cells

**Fig. 4:**
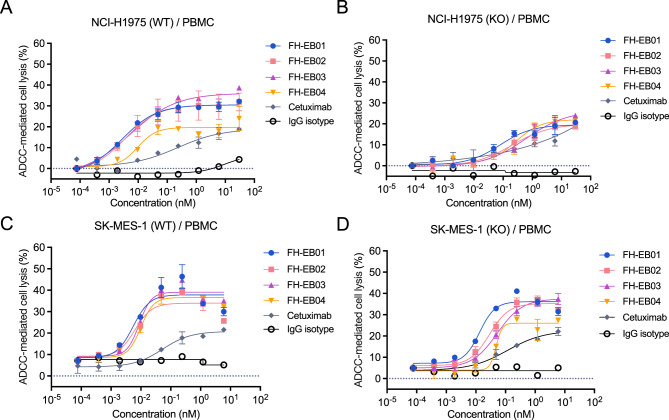
The bsAbs induced significant ADCC effects in NCI-H1975 and SK-MES-1 cells in vitro. **A-D**, dose-dependent cell lysis effects of the FH-EB01, FH-EB02, FH-EB03, FH-EB04, cetuximab and IgG isotype against NCI-H1975 (WT) cells (**A**), NCI-H1975 (KO) cells (**B**), SK-MES-1 (WT) cells (**C**) and SK-MES-1 (KO) cells (**D**). NCI-H1975 (KO) and SK-MES-1 (KO) were B7H3-knockout cell lines. Data were shown as mean ± SD. SD, standard deviation; WT, wide type; KO, B7H3-knockout

To evaluate the ADCC function of the bsAbs, tumor cells were treated with antibodies in the presence of human PBMC. Dose-dependent cell lysis was observed in B7H3 and EGFR co-expressing tumor cells NCI-H1975 when treated with the four bsAbs (Fig. 4A). FH-EB03 induced more potent ADCC in WT cells, showing a maximum lysis of 30.50% [95%CI: 29.09–32.16] and an EC50 of 2.79 [95%CI: 1.31–4.51] pM, as compared to KO cells, which showed a maximum lysis of19.55% [95%CI: 16.96–25.74] and an EC50 of 79.29 [95%CI: 35.60–292.4] pM, repectively. FH-EB01 and FH-EB02 exhibited better effect than cetuximab, and FH-EB04 showed a lower effect similar to cetuximab. When B7H3 was genetically knocked out, all bsAbs show a similar effect and the cell lysis effects were remarkably hampered (Fig. 4B). In the SK-MES-1 (WT) and its B7H3-KO isogenic models, dose-dependent cell lysis was also observed and the lysis behavior induced by the four bsAbs were similar, and the cell lysis effects dependent on B7H3 expression were also detected (Fig. 4C and D). In these experiments, cetuximab induced a weaker ADCC response and exhibited no selectivity between B7H3/EGFR co-expressing cells and EGFR single-positive cells (Fig. 4A–D). Besides, when profiling the immune cells in the control group and the FH-EB02 group in a LUAD patient-derived xenograft (PDX) model, PDX-011, we observed significantly increased infiltration levels of DC cells and NK cells after FH-EB02 administration (Supplemental Figure 5A–G).

### FH-EB02 inhibited the growth of tumors coexpressing EGFR and B7H3

**Fig. 5:**
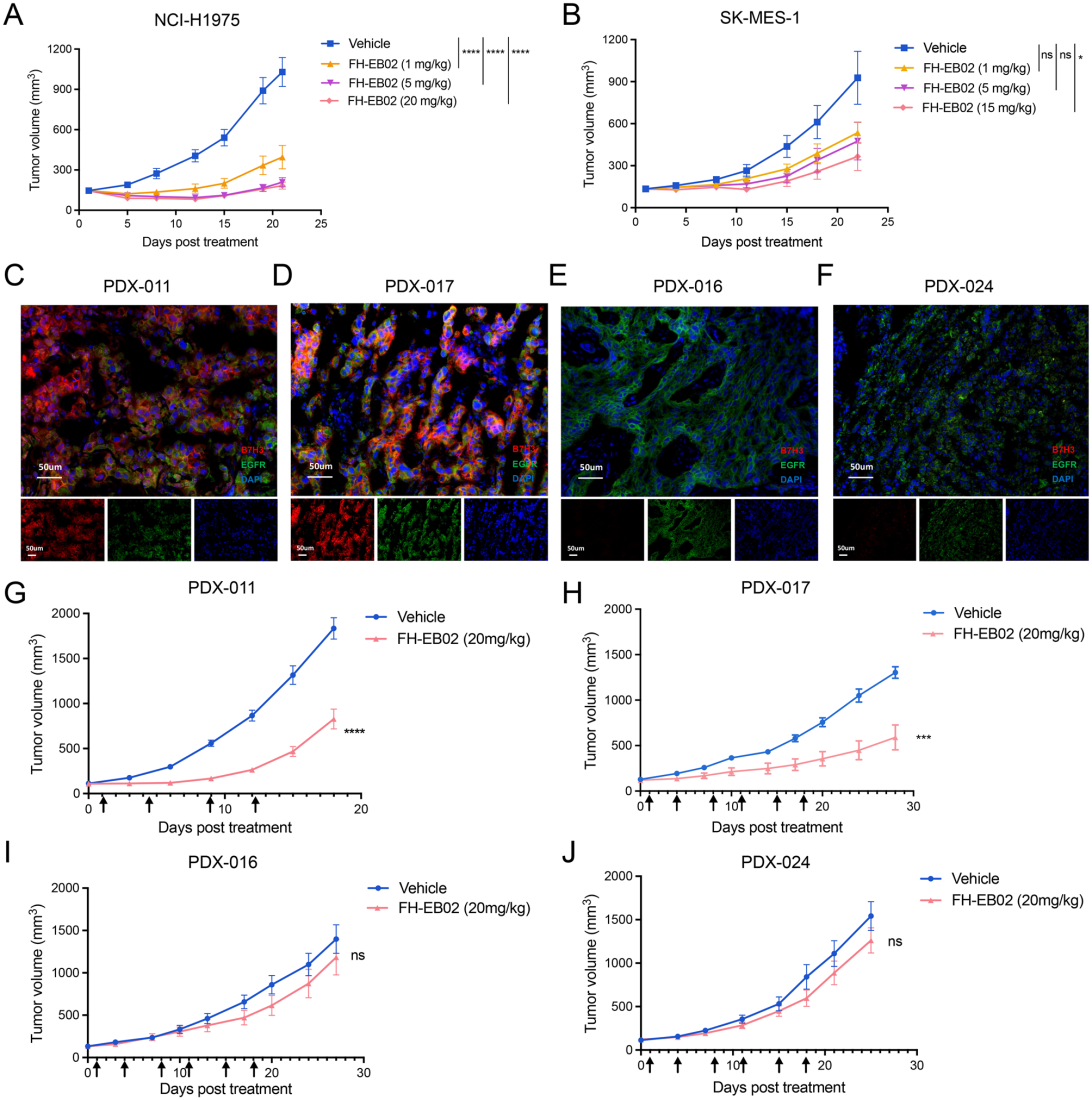
Anti-tumor activity of FH-EB02 in CDX and PDX models. **A** and **B**, Tumor volume over time in NCI-H1975 (**A**) or SK-MES-1(**B**) xenografts treated with FH-EB02 (n=7 per group). **C**-**F**, Immunofluorescence images of B7H3 and EGFR staining in PDX models indicated. **G**-**J**, Tumor volume over time in PDX models PDX-011 (**C**), PDX-017 (**D**), PDX-016 (**E**), and PDX-024 (**F**) treated with FH-EB02. PDX-011 and PDX-017 showed B7H3/EGFR co-expression, whereas PDX-016 and PDX-024 were single-positive for EGFR. Eight mice were used per group for PDX-011, and six for PDX-016, PDX-017, and PDX-024. Tumor volumes were shown as mean ± SEM. ns, non-significance, *P<0.05, ***P<0.001, ****P<0.0001. SEM, standard error of the mean; CDX, cell line-derived xenograft; PDX, patient-derived xenograft

In the in vitro assessments, FH-EB02, which has a weaker anti-EGFR and a more potent anti-B7H3 arm (Fig. 2B, G, H), demonstrated a greater binding capacity with tumor cells (Fig. 3A), higher selectivity for B7H3/EGFR coexpressing tumor cells (Fig. 3G), as well as prominent ADCC effect (Fig. 4A and C). Therefore, we selected FH-EB02 to further evaluate its therapeutic effects in xenograft tumor models. NCI-H1975 or SK-MES-1 cells were implanted into the right flanks of BALB/c nude mice, which were then treated twice a week with FH-EB02 or PBS for three weeks. A rapid decrease in tumor volume was observed in the NCI-H1975 model. At the end of the treatment, FH-EB02 at 1 mg/kg reduced the average tumor volume by 61.58%, 5 mg/kg by 79.71%, and 20 mg/kg by 82.21% (Fig. 5A). In the SK-MES-1 xenograft model, FH-EB02 at 1, 5, and 15 mg/kg reduced the average tumor volume by 42.34%, 48.72%, and 60.61%, respectively, on day 22, with a statistically significant difference compared to the control group at 15 mg/kg (Fig. 5B). To further validate the efficacy of FH-EB02, four PDX models were successful establishment including two B7H3/EGFR co-expression positive (PDX-011 and PDX-017) and two EGFR single-positive (PDX-016 and PDX-024) models (Fig. 5C-J). The co-expression ratio of cells in PDX-011 was 57.61%, similar to PDX-017 of 58.24%. In PDX-016, the co-expression ratio was 2.77%, and that in PDX-024 was 6.70% (Supplemental Figure 5H). In the coexpressing models, PDX-011 (LUAD) and PDX-017 (LUSC), when FH-EB02 was administrated twice a week at 20 mg/kg for two weeks, reductions of 64.5% and 54.7% in tumor volume were observed, respectively (Fig. 5C, D, G, and H). However, in the EGFR single-positive models, the bsAb showed no effect on the tumor growth (Fig. 5E, F, I, and J). FH-EB02 did not cause significant weight loss in all tested mouse models (Supplemental Figure 4A-L). Besides, flow-cytometric analysis of PDX-011 tumors revealed a significant increase in NK and DC cells within the immune microenvironment after FH-EB02 treatment (Supplemental Figure 5A-G).

### FH-EB02 was well tolerated in cynomolgus monkeys 

Previously, cetuximab was reported to be associated with significant on-target toxicity in preclinical models and in clinical practice [11–14]. To further assess the safety profile of FH-EB02, a pilot study was conducted in cynomolgus monkeys, evaluating the drug’s toxicity at weekly doses of 60 mg/kg or 100 mg/kg for four consecutive weeks. We observed increased serum concentrations of FH-EB02 in a dose-dependent manner (Supplemental Figure 6A and B, Supplemental Tables 2 and 3).

In the 60 mg/kg dosing group, the area under the curve from time zero to the last observation (AUC_0-t_) following the initial dose was 47,637 h·μg/mL in male animal and 56,474 h·μg/mL in female animal (Supplemental Table 4). Upon administration of the last dose, the AUC_0-t_ values escalated to 75,938 and 36,840 h·μg/mL for male and female, respectively, corresponding to drug accumulation ratios of 0.65 for female and 1.59 for male (Supplemental Table 4). The accumulation indices were 0.96 and 1.13 for female and male, respectively (Supplemental Table 4). In the 100 mg/kg dosing group, the AUC_0-t_ after the first dose was 75,707 h·μg/mL in the male animal and 76,486 h·μg/mL in the female animal. As for the last dose, the AUC_0-t_ values increased to 123,134 and 115,997 h·μg/mL for the male and the female, respectively, indicating drug accumulation ratios of 1.52 for the female and 1.63 for the male with repeated dosing. The corresponding accumulation indices for C_max_ were 1.54 and 1.08 for the female and the male, respectively. As measured by AUC_0-t_ and C_max_, there was no evident tendency toward accumulation in either the low-dose or high-dose groups of FH-EB02 (Supplemental Table 4), suggesting a low risk of increased systemic exposure and associated toxicities upon repeated dosing.

FH-EB02 demonstrated good tolerability at both dosages, with all animals surviving the study. Apart from soft stools noted in three out of four animals, there were no significant clinical observations, and no skin abnormalities were detected in any of the animals. During the acclimation phase and at specific intervals (days 3, 7, 14, 21, and 29) throughout the dosing period, peripheral blood samples were collected from the animals for analysis (Supplemental Figure 7). Additionally, on day 17, an extra blood collection was performed in the 100 mg/kg group for biochemical assessments. Hematological evaluations included measurements of hemoglobin (Supplemental Figure 7A), white blood cells (Supplemental Figure 7B), and platelets (Supplemental Figure 7C), as well as coagulation parameters, such as prothrombin time, activated partial thromboplastin time, and fibrinogen content (Supplemental Figure 7D-F). These measurements were all within the normal physiological range, without dose- or time-dependent changes observed. Blood biochemistry results were within the normal range, except for a transient and reversible increase in aspartate aminotransferase and alanine aminotransferase levels in one animal from each dosing group, which occurred three days after the first dose and showed a trend towards recovery by day 7 (Supplemental Figure 7G–L). Based on these observations, the maximal tolerated dose was anticipated to be greater than 100 mg/kg in cynomolgus monkeys.

## Discussion

Bispecific antibodies can simultaneously target two abnormally overexpressed antigens, thereby blocking dual signaling pathways that are critical to tumor development [27]. Our study was the first to investigate the co-expression of B7H3 and EGFR in NSCLC, with positive rates exceeding 50% across squamous carcinoma, adenocarcinoma, and EGFR-mutant subtypes. This co-expression correlated significantly with poor prognosis and immunosuppressive tumor microenvironments. Importantly, we identified FH-EB02 from a pool of four bispecific antibodies targeting both antigens. Compared to cetuximab, FH-EB02 exhibited enhanced selectivity and safety through its strong B7H3 binding and moderate EGFR engagement, showing superior EGFR pathway blockade and ADCC effects. These findings demonstrate that FH-EB02 could enhance therapeutic efficacy while mitigating cutaneous toxicity in NSCLC, supporting its potent for further clinical exploration.

Cetuximab and amivantamab have gained indication for the treatment of several solid tumors, but up to 90% of patients experienced a rash with cetuximab and 35% of patients discontinued amivantamab in the MARIPOSA study, which severely restricts their clinical utility [8, 28]. The limited selectivity of EGFR antibodies drove on-target toxicity in normal skin tissues, necessitating the development of alternative strategy such as bsAbs with enhanced specificity to improve safety [29]. As we know, B7H3 was only overexpressed in cancer cells [15, 26, 30], and the associated ADC has become a remarkable therapy in SCLC [31]. Therefore, we firstly investigated EGFR/B7H3 co-expression in a large NSCLC cohort (*n* = 222), and found high positivity rates across key subtypes: 75.9% in untreated LUSC, 55.0% in EGFR/ALK/ROS1 wild-type LUAD, and 53.3% in EGFR-TKI-resistant LUAD. More importantly, consistent with previous reports [6, 32, 33], we found that patients with both B7H3 and EGFR overexpression had inferior efficacy of frontline therapy and poor prognosis. These patients also exhibited an immunosuppressive microenvironment, suggesting that an unmet need remains for this sub-population in the clinical setting.

Our study engineered four EGFR/B7H3 bsAbs with differential binding affinities, all of which demonstrated superior tumor cell binding, EGFR pathway inhibition, and ADCC activity compared to cetuximab. Critically, tumor targeting was B7H3-dependent, and the binding capacity was markedly decreased upon B7H3 knockout, confirming tumor cell-selective cytotoxicity. Furthermore, the lead candidate FH-EB02 demonstrated optimal efficacy-selectivity balance and robust tumor suppression in EGFR/B7H3-coexpressing CDX and PDX models. Safety evaluation in cynomolgus monkeys revealed only transient aspartate aminotransferase elevation without cutaneous toxicity, highlighting its clinical potential as a prioritized therapy for NSCLC. Similarly, EGFR/B7H3-targeted bsAbs or ADCs, including IBI334 and IBI3001 from Innovent Biologics [34, 35], are currently in Phase I/II clinical trials with results pending (NCT05774873, NCT06349408). This suggests that dual inhibition of EGFR and B7H3 might be an important strategy for patients with NSCLC in the future.

Additionally, we explored the mechanisms underlying the efficacy of FH-EB02. In terms of antitumor activity, similar to other bsAbs, FH-EB02 also exhibited dual antitumor mechanisms: direct growth inhibition via EGFR phosphorylation blockade and indirect immune activation through the ADCC function [36, 37]. To amplify the ADCC activity, we engineered the antibody’s Fc domain (S239D/I332E mutations in IgG1 CH2), enhancing FcγRIIIa binding on NK cells and potentiating their cytotoxic function. For the safety exploration of FH-EB02, it was observed that the blockade to EGF significantly decreased after B7H3 knockout, with exceeding 40-fold IC50 ratio (KO/WT) in NCI-H1975 cells. This implied that FH-EB02 primarily bound to tumor cells and blocked EGFR signaling through its interaction with B7H3, providing a large therapeutic window and thereby further reducing the binding of the bsAb to EGFR targets in normal tissues and mitigating side effects.

This study was subject to several limitations. Firstly, although we assessed EGFR/B7H3 co-expression in a large cohort encompassing both driver-negative and EGFR-TKI-resistant NSCLC, other genomic subgroups and Caucasian populations remained understudied. Secondly, the heterogeneity of tumor cells may lead to expression of the two markers in different cells, resulting in some false-positive results [38]. Besides, although FH-EB02 demonstrated superior selectivity and safety over cetuximab in preclinical models, its efficacy and safety in the patients remained unvalidated. Moreover, previous studies had shown that B7H3 can upregulate IL-2, IL-6, IL-17, and TGF-β1 levels while diminishing IFN-γ production [39], and B7H3 antibodies can promote the infiltration of CD8^+^ T cells in the tumor microenvironment [17, 18]. B7H3 was highly expressed in small cell lung cancer [40], and ADC based on B7H3 had shown significant efficacy [41, 42]. Our research indicated that B7H3 was also highly expressed in NSCLC. Therefore, with a promising application in NSCLC, B7H3-based drugs, such as oncolytic viruses, B7H3 blockades, ADCs, have shown pretty good antitumor efficacy accompanied by an increased number of CD8^+^ TILs, M1-polarized macrophages and immune cell reactivation [26, 43, 44]. Although FH-EB02 enhanced NK/DC infiltration in the tumor microenvironment, the humanized design prevented comprehensive immune profiling in immunocompetent models, and the effects on adaptive immunity (T/B cells) remained unknown.

## Conclusions

EGFR and B7H3 are highly co-expressed in NSCLC. FH-EB02, a bsAb that binds to cells through B7H3 and subsequently blocks the EGFR signaling pathway, has demonstrated promising efficacy in EGFR ligand blockade, ADCC cell killing in vitro, and pharmacological and toxicity evaluations in vivo. Based on these findings, FH-EB02 is planned to enter clinical trials targeting advanced NSCLC characterized by co-expression of B7H3 and EGFR.

## Electronic supplementary material

Below is the link to the electronic supplementary material.

Supplementary Material 1

## Data Availability

The datasets used during the current study are available from the corresponding author on reasonable request.

## Abbreviations

EGFR: Epidermal growth factor receptor
B7H3: B7 homolog 3 protein
NSCLC: Non-small cell lung cancer
BsAb: Bispecific antibody
ADCC: Antibody-dependent cell-mediated cytotoxicity
CDX: Cell line-derived xenograft
PDX: Patient-derived xenograft
PFS: Progression-free survival
OS: Overall survival
ADC: Antibody-drug conjugates
SCLC: Small cell lung cancer
RT: Room temperature
ABC: Antibody binding capacity
MESF: Molecules of equivalent soluble fluorochrome
LUSC: Lung squamous cell carcinoma
LUAD: Lung adenocarcinoma
KO: Knockout
WT: Wide type
