# Supplementary material for: Development of a bispecific antibody that inhibits EGFR and B7H3 in NSCLC
Source: Biomark Res. 2025 Nov 25;13:158. doi: 10.1186/s40364-025-00872-1 (PMC12750570; doi:10.1186/s40364-025-00872-1)
Supplement: Supplementary file 1 — Supplementary Material 1 [file 40364_2025_872_MOESM1_ESM.docx]

**Supplemental material**

**Development of a bispecific antibody that inhibits EGFR and B7H3 in NSCLC**

**Authors:** Xinxin Zhi^1#^, Jiale Wang^1#^, Junhong Guo^2#^, Libo Luo^1^, Hui Sun^1^, Yi Li^3^, Zhen Zhao^3^, Chenghu Wang^3^, Lifang Zhu^3^, Xi Li^3^, Feng Wang^3^, Fei Li^4^, Kebing Yu^3*^, Shengxiang Ren^1*^

^#^ Xinxin Zhi, Jiale Wang, and Junhong Guo contributed equally to this study.

*Corresponding authors:

Shengxiang Ren, Department of Medical Oncology, Shanghai Pulmonary Hospital, School of Medicine, Tongji University, Shanghai, 200433, China. Tel: +86-21-65115006; E-mail: harry_ren@tongji.edu.cn

Kebing Yu, Shanghai Fuhong Biopharmaceutical Co., Ltd, Shanghai, 200120, China. E-mail: kebing.yu@fuhongpharma.com

**Supplemental Methods:**

**Affinity measurements of bsAbs arms**

Anti-EGFR binding affinity was determined using Octet R2 Biolayer Interferometry (Sartorius, Germany). Briefly, anti-EGFR scFv fused to human IgG1 Fc was immobilized on AHC biosensor until signal reached 1 nm. Recombinant EGFR protein (Acro Biosystems, EGR-H5222, China) was loaded on the immobilized antibody for 120 seconds and then dissociated for 100 seconds. Association and dissociation signals were monitored to calculate kinetic parameters by Octet Analysis Studio (Version 12.2.0.20). Anti-B7H3 binding affinity was measured using surface plasmon resonance (SPR)-based Biacore T200 (Cytiva, USA). Anti-B7H3 antibodies with human IgG1 Fc were captured by CM5 sensor chip (GE Healthcare, USA) pre-immobilized with anti-human IgG antibody. Multiple concentrations of human B7-H3 protein (Acro Biosystems, B73-H82E6, China) were sequentially loaded on the captured antibody for 120 seconds and then dissociated for 300 seconds, which was recorded using Biacore control Software (Version 3.2.1). Association and dissociation signals were monitored to calculate kinetic parameters by Biacore T200 Evaluation Software (Version 3.2.1).

**Immunohistochemistry**

A total of 222 paraffin-embedded tissues were cut into 4–5 µm thick sections and placed on glass slides for subsequent staining. After routine dewaxing in xylene and hydration in ethanol, antigen retrieval was performed. Slides were immersed in citrate buffer (pH 6.0) (Servicebio, G1202, China) and heated in a microwave oven for 15 min for heat-induced antigen retrieval. After cooling at room temperature for 20 min, the sections were treated with 3% H₂O₂ (Servicebio, G0115, China) for 25 min to quench endogenous peroxidase activity. Following three washes with phosphate-buffered saline (PBS) (Servicebio, G0002, China), the sections were blocked with 3% BSA (Servicebio, G5001, China) at room temperature for 30 min to prevent non-specific binding. The slides were then stained with primary antibodies against EGFR (Dako, M3563, 1:100, Denmark), B7H3 (Cell signaling Technology, clone D9M2L #14058, 1:100, USA), CD3 (Dako, IR50361-2CN, Denmark), CD8 (Dako, IR62361-2CN, Denmark), CD68 (Dako, IR61361-2CN, Denmark), Foxp3 (BioLegend, Clone 206D, 1:200, USA) at 4 °C overnight, respectively. For the negative control, the primary antibody was replaced with isotype-matched irrelevant IgG under the same condition. After incubation with a secondary antibody (Servicebio, GB23301, China) for one hour at 37 °C, the slides were washed by PBS for three times and the signal was revealed by the DAB Kit (Servicebio, G1211, China). Subsequently, the sections were immersed in hematoxylin (Servicebio, G1004, China) solution and counterstained for 1 - 2 minutes at room temperature. Finally, after mounting with neutral resin, the sections were observed under an optical microscope (Zeiss, Axio Scope A1, Germany). The number of positive immune cells was recorded under a 200× objective lens. The staining intensity of B7-H3 and EGFR was categorized into four levels: 0, 1+, 2+, and 3+.

**Flow cytometry**

For analysis of immune microenvironment, fresh tissues were dissociated into single-cell suspensions using the mouse tumor dissociation kit (Miltenyi Biotec, 130-096-730, Germany) according to the manufacturer's instructions. The cell suspension was filtered through a 70 µm filter mesh, and the pellet was collected by centrifugation at 500g for 5 minutes at 4°C. The pellet was lysed with erythrocyte lysis buffer (Tiangen, GRT122-02, China) at room temperature (RT) for 5 minutes, washed twice with PBS (Biosharp, BL30-2A), then washed once with DPBS (Biosharp, BL310-A, China). Fixable viability dye (Thermo, 65-2860-40, USA) was added and the cells were stained in the dark at RT for 15 minutes. After one wash with stain buffer (BD bioscience, 554657, USA), anti-mouse CD16/CD32 (BD Pharmingen, 553142, USA) was added for 10 minutes of staining at RT, followed by the addition of fluorescence-conjugated cell membrane surface antibodies CD45 (BD Pharmingen, 557659, USA), CD11b (Biolegend, 101233, USA), IA-IE (BD Pharmingen, 746669, USA), CD11C (BD Pharmingen, 563048, USA), NKp46 (BD Pharmingen, 564069, USA), and F480 (BD Pharmingen, 743282, USA), CD86 (BD Pharmingen, 558703, USA) for 20 minutes of staining. After fixation and permeabilization with a permeabilization agent (BD Pharmingen, 562574, USA), PE fluorescence-conjugated CD206 (BD Pharmingen, 568273, USA) nuclear antibodies were added. Finally, the samples were analyzed on flow cytometer (Cytek Aurora, Shanghai, China) and analyzed using FlowJo (Version 10.9.0).

**Supplemental Results**

**Supplemental Figure 1. Co-expression of B7H3 and EGFR and its correlation with immune infiltration.**

A and B, Representative images of immunohistochemical staining illustrating the grading of B7H3 and EGFR expression. C-H, The distribution of expression levels of B7H3 or EGFR in patients with untreated squamous cell carcinoma, untreated adenocarcinoma, or EGFR TKI-resistant adenocarcinoma. I and J, Representative images of immunohistochemical staining for CD3 (I) and CD68 (J) in specimens with negative or positive co-expression of B7H3 and EGFR. K and L, Statistics analyses of the data in (I) and (J), respectively. A, B, and J were from lung squamous cell carcinoma, and I was from lung adenocarcinoma. ns, non-significance.

**Supplemental Figure 2. Impact of co-expression of B7H3 and EGFR on patient survival.**

A-C, Impact of B7H3 and EGFR co-expression on progression-free survival in patients with untreated squamous cell carcinoma (A), untreated adenocarcinoma (B), and EGFR TKI-resistant adenocarcinoma (C). D-F, Impact of B7H3 and EGFR co-expression on overall survival in patients with untreated squamous cell carcinoma (D), untreated adenocarcinoma (E), and EGFR TKI-resistant adenocarcinoma (F).

**Supplemental Figure 3.** **Receptor densities of B7H3 and EGFR，as well as EGFR phosphorylation inhibition by bispecific antibodies**

**A, Receptor densities of B7H3 and EGFR on A431, NCI-H1975, and SK-MES-1 cells. B,** Inhibition of the EGF-stimulated EGFR phosphorylation by FH-EB01, FH-EB02, FH-EB03 and cetuximab in SK-MES-1 cells, NCI-H1975 and A431 cells.

**Supplemental Figure 4. Body-weight changes in mice during the anti-tumor treatment with FH-EB02.**

A-F, Changes in total body weight of indicated mice during the treatment. G-L, Changes in relative body weight of indicated mice during the treatment. PDX-011 and PDX-017 were B7H3/EGFR co-expression positive, derived from lung adenocarcinoma and lung squamous cell carcinoma, respectively. PDX-016 and PDX-024 were EGFR single positive, from lung squamous cell carcinoma and lung adenocarcinoma, respectively. The body weight of mice did not change significantly in any of the four PDX models after FH-EB02 treatment. ns, non-significance.

**Supplemental Figure 5. Changes in the tumor microenvironment following FH-EB02 treatment in PDX-011.**
A, Flow cytometry gating strategy for NK and DC cells. B, Individual flow cytometry plots of NK cells in mice from the vehicle (upper) and FH-EB02 treatment (lower) groups. C, Individual flow cytometry plots of DC cells in mice from the vehicle (upper) and FH-EB02 treatment (lower) groups. D and E, Representative plots of NK (D) and DC (E) cells. F and G, Bar plots showing the statistical analysis of NK (F) and DC (G) cells. H, The co-expression ratio of cells in the four PDX models. ****P*<0.001.

**Supplemental Figure 6. Pharmacokinetics profile of FH-EB02 in cynomolgus monkeys.**

A. Serum drug concentration changes of FH-EB02 in cynomolgus monkeys following initial intravenous administration on day 1. B, Changes in serum drug concentration following the fourth administration of FH-EB02 on Day 22. Serum was collected at each of the following time points: immediately before, and at 10 min, 2 h, 6 h, 24 h, 48 h, 72 h, 120 h and 168 h after the FH-EB02 administration.

**Supplemental Figure 7. Continuous hematological monitoring in cynomolgus monkeys following** **FH-EB02 administration.**

A-L, Hemoglobin (A), WBC (B), PLT (C), PT (D), APTT (E), FIB (F), ALT (G), AST (H), TBIL (I), ALB (J), GLOB (K), and A/G (L) changes over time following FH-EB02 administration in cynomolgus monkeys. WBC, white blood cells; PLT, platelet; PT, prothrombin time; APTT, activated partial thromboplastin time; FIB, fibrinogen; ALT, alanine aminotransferase; AST, aspartate aminotransferase; TBIL, total bilirubin; ALB: albumin; GLOB, globulin.

Supplemental Table 1. Clinical characteristics and immunohistochemical distribution of B7H3 and EGFR in patients with advanced NSCLC.

|  | Total  (N=222) | Squamous cell carcinoma (untreated)  (N=87) | Adenocarcinoma (untreated)  (N=60) | Adenocarcinoma (EGFR-TKI resistant)  (N=75) |
| --- | --- | --- | --- | --- |
| Gender |  |  |  |  |
| Male | 176 (79.3%) | 85 (97.7%) | 51 (85.0%) | 40 (53.3%) |
| Female | 46 (20.7%) | 2 (2.3%) | 9 (15.0%) | 35 (46.7%) |
| Age (years) |  |  |  |  |
| Mean (SD) | 64.0 (9.13) | 65.7 (6.87) | 64.5 (7.98) | 61.7 (11.6) |
| ECOG-PS |  |  |  |  |
| 0-1 | 195 (87.8%) | 82 (94.3%) | 54 (90.0%) | 59 (78.7%) |
| 2 | 27 (12.2%) | 5 (5.7%) | 6 (10.0%) | 16 (21.3%) |
| Smoking habit |  |  |  |  |
| No | 137 (61.7%) | 39 (44.8%) | 33 (55.0%) | 63 (84.0%) |
| Yes | 87 (39.2%) | 48 (55.2%) | 27 (45.0%) | 12 (16.0%) |
| Stage |  |  |  |  |
| III | 58 (26.1%) | 43 (49.4%) | 13 (21.7%) | 2 (2.7%) |
| IV | 164 (73.9%) | 44 (50.6%) | 47 (78.3%) | 73 (97.3%) |
| PD-L1 |  |  |  |  |
| <1% | 99 (44.6%) | 36 (41.4%) | 27 (45.0%) | 36 (48.0%) |
| 1-49% | 47 (21.2%) | 20 (23.0%) | 10 (16.7%) | 17 (22.7%) |
| >=50% | 53 (23.9%) | 17 (19.5%) | 18 (30.0%) | 18 (24.0%) |
| Missing | 23 (10.4%) | 14 (16.1%) | 5 (8.3%) | 4 (5.3%) |
| B7H3 grade |  |  |  |  |
| 0 | 34 (15.3%) | 11 (12.6%) | 7 (11.7%) | 16 (21.3%) |
| 1+ | 74 (33.3%) | 25 (28.7%) | 30 (50.0%) | 19 (25.3%) |
| 2+ | 48 (21.6%) | 18 (20.7%) | 12 (20.0%) | 18 (24.0%) |
| 3+ | 66 (29.7%) | 33 (37.9%) | 11 (18.3%) | 22 (29.3%) |
| EGFR grade |  |  |  |  |
| 0 | 59 (26.6%) | 13 (14.9%) | 23 (38.3%) | 23 (30.7%) |
| 1+ | 57 (25.7%) | 35 (40.2%) | 4 (6.7%) | 18 (24.0%) |
| 2+ | 45 (20.3%) | 19 (21.8%) | 12 (20.0%) | 14 (18.7%) |
| 3+ | 61 (27.5%) | 20 (23.0%) | 21 (35.0%) | 20 (26.7%) |

Supplemental Table 2. The pharmacokinetic profile of FH-EB02 following the first administration in cynomolgus monkeys

| Time (h) | 60 mg/kg ((ng/mL) | | 100 mg/kg (ng/mL) | |
| --- | --- | --- | --- | --- |
|  | Male | Female | Male | Female |
| 0 | BQL | BQL | BQL | BQL |
| 0.667 | 1312976 | 1400227 | 2033106 | 1866718 |
| 2.5 | 1120019 | 1178121 | 1528083 | 1624651 |
| 6.5 | 851654 | 1015444 | 1291559 | 1537601 |
| 24.5 | 502585 | 513693 | 751932 | 699824 |
| 48.5 | 323669 | 360202 | 473498 | 465301 |
| 72.5 | 215516 | 275651 | 357348 | 346458 |
| 120.5 | 130527 | 181186 | 242423 | 253618 |
| 168.5 | 76919 | 124397 | 164122 | 148973 |

BQL: Below the Quantification Limit. Linear range: 200 to 6400 ng/mL.

Supplemental Table 3. The pharmacokinetic profile of FH-EB02 following the last administration in cynomolgus monkeys

| Time (h) | 60 mg/kg((ng/mL) | | 100 mg/kg((ng/mL) | |
| --- | --- | --- | --- | --- |
|  | Male | Female | Male | Female |
| 0 | 166813 | 11253 | 258098 | 257157 |
| 0.667 | 1436595 | 1342453 | 2202220 | 2868359 |
| 2.5 | 1482637 | 1232354 | 2135512 | 2488493 |
| 6.5 | 1045467 | 994974 | 1753609 | 1921096 |
| 24.5 | 771920 | 543768 | 1100973 | 1080487 |
| 48.5 | 520537 | 252045 | 831681 | 694783 |
| 72.5 | 409298 | 134137 | 693313 | 550935 |
| 120.5 | 247890 | 11251 | 433553 | 406745 |
| 168.5 | 159575 | 647 | 303284 | 281552 |

Supplemental Table 4. Toxicodynamics of FH-EB02 after repeated administration in cynomolgus monkeys.

| Group | Dosing day | Gender | T_max_ (h) | C_max_ (ug/mL) | AUC_0-t_ (h.ug/mL) |
| --- | --- | --- | --- | --- | --- |
| 60 mg /kg | Day 1 | Male | 0.667 | 1313 | 47637 |
|  |  | Female | 0.667 | 1400 | 56474 |
|  | Day 22 | Male | 2.5 | 1483 | 75938 |
|  |  | Female | 0.667 | 1342 | 36840 |
| 100 mg/kg | Day 1 | Male | 0.667 | 2033 | 75707 |
|  |  | Female | 0.667 | 1867 | 76486 |
|  | Day 22 | Male | 0.667 | 2202 | 123134 |
|  |  | Female | 0.667 | 2868 | 115997 |
